# Single cell oil of oleaginous fungi from the tropical mangrove wetlands as a potential feedstock for biodiesel

**DOI:** 10.1186/1475-2859-11-71

**Published:** 2012-05-30

**Authors:** Mahesh Khot, Srijay Kamat, Smita Zinjarde, Aditi Pant, Balu Chopade, Ameeta RaviKumar

**Affiliations:** 1 Institute of Bioinformatics and Biotechnology, University of Pune, Pune 411 007, India; 2 Department of Biotechnology, University of Pune, Pune 411 007, India

**Keywords:** Mangrove wetlands, Oleaginous fungi, Single cell oil, Fatty acid methyl ester, *Aspergillus terreus*

## Abstract

**Background:**

Single cell oils (SCOs) accumulated by oleaginous fungi have emerged as a potential alternative feedstock for biodiesel production. Though fungi from mangrove ecosystem have been reported for production of several lignocellulolytic enzymes, they remain unexplored for their SCO producing ability. Thus, these oleaginous fungi from the mangrove ecosystem could be suitable candidates for production of SCOs from lignocellulosic biomass. The accumulation of lipids being species specific, strain selection is critical and therefore, it is of importance to evaluate the fungal diversity of mangrove wetlands. The whole cells of these fungi were investigated with respect to oleaginicity, cell mass, lipid content, fatty acid methyl ester profiles and physicochemical properties of transesterified SCOs in order to explore their potential for biodiesel production.

**Results:**

In the present study, 14 yeasts and filamentous fungi were isolated from the detritus based mangrove wetlands along the Indian west coast. Nile red staining revealed that lipid bodies were present in 5 of the 14 fungal isolates. Lipid extraction showed that these fungi were able to accumulate > 20% (w/w) of their dry cell mass (4.14 - 6.44 g L^-1^) as lipids with neutral lipid as the major fraction. The profile of transesterified SCOs revealed a high content of saturated and monounsaturated fatty acids i.e., palmitic (C16:0), stearic (C18:0) and oleic (C18:1) acids similar to conventional vegetable oils used for biodiesel production. The experimentally determined and predicted biodiesel properties for 3 fungal isolates correlated well with the specified standards. Isolate IBB M1, with the highest SCO yield and containing high amounts of saturated and monounsaturated fatty acid was identified as *Aspergillus terreus* using morphotaxonomic study and 18 S rRNA gene sequencing. Batch flask cultures with varying initial glucose concentration revealed that maximal cell biomass and lipid content were obtained at 30gL^-1^. The strain was able to utilize cheap renewable substrates *viz*., sugarcane bagasse, grape stalk, groundnut shells and cheese whey for SCO production.

**Conclusion:**

Our study suggests that SCOs of oleaginous fungi from the mangrove wetlands of the Indian west coast could be used as a potential feedstock for biodiesel production with *Aspergillus terreus* IBB M1 as a promising candidate.

## Background

Biodiesel as an alternative fuel has been in the forefront of the liquid biofuel sector for the last two decades. The use of edible vegetable oils such as soybean, rapeseed and non-edible oils such as *Jatropha* in the United States, Europe and India, respectively, as oil feedstock for biodiesel production needs to be augmented with newer technologies. To meet the demand of the biodiesel industry, alternative oil sources are being explored and developed. Recently, microbial lipids (single cell oils, SCOs) accumulated by oleaginous microorganisms e.g., microalgae and fungi, with 20% or more of their cell mass being composed of lipids, have emerged as a potential feedstock for biodiesel production [1,2].

The applications of oleaginous fungi for biodiesel are very few although they have several advantages over conventional plant and algal resources as they can be easily grown in bioreactors, have short life cycles, display rapid growth rates, are unaffected by space, light or climatic variations, are easier to scale up and have the ability to utilize a wide range of inexpensive renewable carbon sources such as lignocellulosic biomass and agro-industrial residues. Rumbold *et al* (2009) have evaluated the utilization of lignocellulosic biomass hydrolysates by two yeasts (*Saccharomyces cerevisiae* and *Pichia stipitis*) and two fungi (*Aspergillus niger* and *Trichoderma reesei*) as feedstock for various industrial fermentations [3]. Several studies reporting lipid accumulation by oleaginous yeasts and filamentous fungi on different renewable substrates such as glycerol, sewage water, whey and molasses has also been reviewed [4]. More recently, metabolic engineering of *Acinetobacter baylyi* ADP1 for improved production of triacylglycerol, a source of biofuel has been studied [5].

Ramos *et al.* (2009) have shown that the biodiesel quality depends upon the fatty acid composition of the oil feedstock [6]. For an oleaginous microbe to be considered as a suitable feedstock for biodiesel, the total lipid content (>20%) and the type of fatty acids (long chain saturated and/or monounsaturated fatty acids) are important criteria. Lipid content and fatty acid composition of SCOs varies in response to environmental factors such as type of carbon source, pH, temperature and according to the nature of microorganism i.e., it is species and strain- specific [4,7]. This is evident from the studies on the psychrophilic oleaginous yeast *Rhodotorula glacialis* wherein both glucose concentration and temperature influenced the composition and degree of unsaturation of fatty acids [8]. In *Mortierella* species, *M. alpina* was able to produce 40% (w/w) SCO while *M. isabellina* ATHUM 2935 gave 50.4% (w/w) oil [9,10] when grown on glucose. The lipid production (w/w wet biomass) in n-hexadecane- and glucose-grown cells were 4.43 and 0.62%, respectively, by *Aspergillus terreus* MTCC 6324 [11]*.* Consequently, since the accumulation of lipids by oleaginous fungi varies, not all oleaginous fungal cells can be used as a feedstock for biodiesel production. Therefore, careful selection of the oleaginous strains of the fungal species and characterization of lipid composition need to be performed to ascertain their suitability for biodiesel production.

Mangrove wetlands constitute specific regions in tropical and subtropical intertidal areas of the world where salt tolerant mangrove plants occur. Fungi from mangrove ecosystem are the second largest group amongst the marine fungi [12]. Being a detritus based habitat, fungi from mangroves play a significant role in the decomposition processes, nutrient cycling and energy flow of the marine web. Although mangrove fungi have been reported for production of several lignocellulolytic enzymes [13], to the best of our knowledge, there are no reports on these marine fungi being explored for their SCO producing capability. In fact, such oleaginous fungi with their battery of lignocellulolytic enzymes could be economically relevant in the production of biodiesel from cheap renewable carbon sources. In this study, a number of fungi were isolated from three different mangrove wetland locations along the Indian west coast. The potential of the fungal cell mass of these isolates was evaluated for their lipid content and SCO profiles for biodiesel production. Fatty acid composition and some physico-chemical properties of the transesterified SCOs (biodiesel) were analyzed in order to ascertain their potential suitability as a fuel.

## Results and discussion

### Isolation of fungi from mangrove wetlands

Fourteen fungal isolates with different visible colony and cell morphology (as seen under the microscope) were obtained from mangrove wetlands, of which 3 were yeasts, while 11 were filamentous fungi.

### Nile red staining

Oleaginous fungi accumulate high levels of lipids when carbon is in excess and a key nutrient such as nitrogen or phosphorous is limiting [14]. These accumulated lipids or SCOs get deposited as intracellular lipid bodies (LBs) which can easily be detected by a fluorescent probe, Nile red. Kimura *et al* (2004) have visualized LBs using this approach, in oleaginous fungi *viz*., *Lipomyces starkeyi* IFO-10381, *Rhodosporidium toruloides* IFO-0559, *Cryptococcus curvatus* IFO-1159, *Mortierella isabellina* IFO7884, *M.nana* IFO-8794, *M.ramanniana var. angulispora* IFO 8187 wherein LBs varying in size, number and shape have been detected [15].

**Figure 1:**
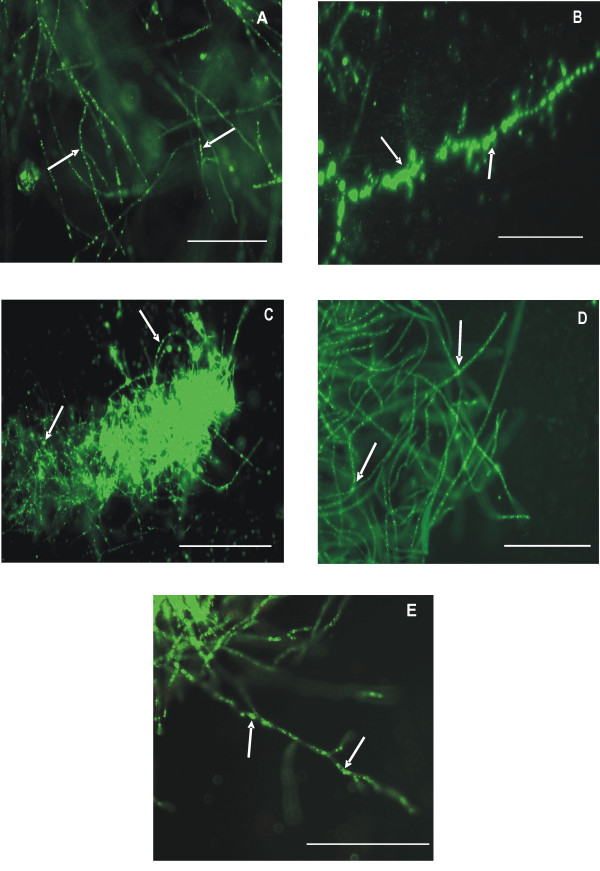
**Microscopic photographs of Nile red fluorescence of fungal isolates from mangrove wetlands.A**) IBB B2; **B**) IBB F14; **C**) IBB G4; **D**) IBB G5; **E**) IBB M1. In the photomicrographs, the intracellular lipid bodies of hyphal cells showing fluorescence after Nile red staining are indicated by white arrows. Bar indicates 50 μm.

In the present study, 14 fungal mangrove isolates when grown under carbon rich and nitrogen limiting conditions, revealed a variable number of oval and ellipsoidal LBs in five fungal strains by fluorescence microscopy (Figure 1). None of the yeast strains isolated exhibited fluorescence, indicating their inability to accumulate lipids under the given growth conditions. All the five lipid accumulating isolates *viz.*, IBB B2, IBB F14, IBB G4, IBB G5 and IBB M1 exhibiting filamentous morphology were selected for quantification of total cellular lipids (SCOs).

### Biomass and total lipid (SCOs) yields

All the 5 fungi grew well in 72 h and showed good biomass under the given carbon rich, nitrogen limiting conditions. The dry cell mass obtained was in the range of 4.1 to 6.44 gL^-1^ for all 5 fungal isolates (Table 1). The total lipid from the cell mass was extracted and estimated (Table 1). All the five fungi were found to be oleaginous, as the total lipid contents were more than 20% (w/w) of dry biomass. IBB M1 showed maximum lipid content of 54% of its dry weight of biomass (0.54 g g^-1^) and also the maximum lipid yield of 2.98 g L^-1^. For the other isolates, the total lipid content varied in the range of 0.25 g g^-1^ to 0.3 g g^-1^ (25 to 30% ,w/w) while the lipid yields were in the range of 1.0 to 1.93 g L^-1^ when extraction was carried out with liquid nitrogen. Comparatively lower amounts of total lipid content and yields were recovered when SCO extractions were carried out using bead beater (Table 1). Thus, higher yields of lipid obtained with liquid nitrogen suggest better extraction efficiency, indicating as suggested by earlier studies that choice of extraction method is critical in efficient recovery of oils from oleaginous fungi [16,17].

**Table 1:** Biomass and SCO yields of oleaginous fungal isolates from mangrove wetlands of Indian west coast

**Isolate**	**Morphology**	**Biomass (CDW ing L**^**-1**^)^*^	**Total lipid**^**#**^**(g g**^**-1**^of CDW)^*^		**Lipid yield**^**#**^**( g L**^**-1**^**)**	
			**Bead beater**	**Liquid nitrogen**	**Bead beater**	**Liquid nitrogen**
IBB B2	Mycelial	4.27 ± 0.1^a^	0.11 ± 0.02^ab^	0.25 ± 0.01^a^	0.47 ± 0.02^a^	1.07 ± 0.1^a^
IBB F14	Mycelial	4.14 ± 0.3^a^	0.15 ± 0.02^ab^	0.28 ± 0.01^a^	0.62 ± 0.01^a^	1.16 ± 0.3^ab^
IBB G4	Mycelial	5.84 ± 0.5^bc^	0.17 ± 0.04^b^	0.30 ± 0.05^a^	0.99 ± 0.02^b^	1.75 ± 0.2^bc^
IBB G5	Mycelial	6.44 ± 0.3^c^	0.09 ± 0.01^a^	0.30 ± 0.03^a^	0.57 ± 0.02^a^	1.93 ± 0.2^c^
IBB M1	Mycelial	5.52 ± 0.2^b^	0.10 ± 0.01^a^	0.54 ± 0.03^b^	0.552 ± 0.3^a^	2.98 ± 0.3^d^

^*^ CDW - cell dry weight determined after 72 h.

^#^ Lipid extractions carried out by two different cell lysis techniques as mentioned in Methods.

The data are shown as the mean ± standard deviation (n = 3). ^a,b,c^ Mean values within a column with different superscript letters differ significantly and were determined statistically as mentioned in Methods (one way ANOVA, Tukey, p < 0.05). Separate analysis was done for each column.

Biomass and SCO yields of the other oleaginous filamentous fungi studied for biodiesel production have been found to be 4.17 g L^-1^ in 96 h and 13.6 g L^-1^ in 48 h with a lipid productivity of 23% and 23.3% for *Mucor circinelloides* MU241, and *Aspergillus sp*. respectively, while a SCO yield of 53% (w/w) of dry cell mass was obtained for *Mortierella isabellina*, when grown on glucose with concentrations ranging from 2% to 7% [7,18,19]. *Aspergillus terreus* MTCC 6324 exhibited 4.43% of lipid productivity when grown on n- hexadecane [11].

### Lipid fractionation

**Figure 2:**
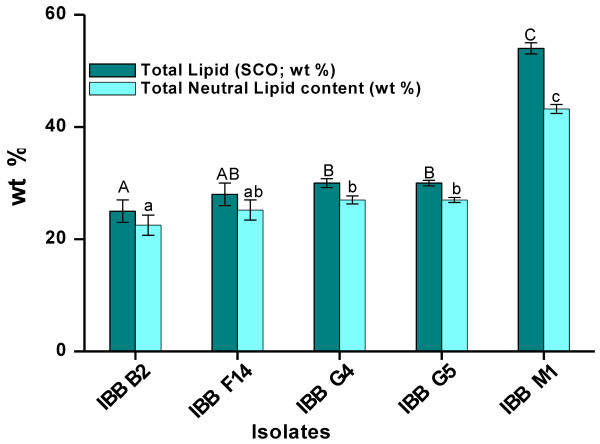
**Total lipid and neutral lipid contents of oleaginous fungi from tropical mangrove wetlands.** The data are expressed as mean ± standard deviation (n = 3). ^A, B, C^ Mean values for total lipid content (wt %) with different superscript letters differ significantly (p < 0.05) and were determined as mentioned in Methods. ^a,b,c^ Mean values for neutral lipid content (wt %) with different superscript letters differ significantly (p < 0.05) and were determined as mentioned in Methods.

The extracted SCOs of five oleaginous mangrove fungal isolates were fractionated by silicic acid column chromatography to determine the neutral lipid content since it includes triacylglycerols (TAGs), one of the major components desirable for biodiesel. The neutral lipid fraction was found to be in the range 22.5 to 43.2% (wt%) of 25 to 54% total lipids of all 5 fungal isolates, respectively (Figure 2). These values correspond to neutral lipid contents of 90% (w/w) of total lipids for 4 isolates IBB B2, F14, G4 and G5 and 80% for IBB M1, indicating that the major fraction of SCO in the cells of these mangrove isolates is neutral lipid. Oleaginous filamentous fungi with potential for biodiesel production such as *Cunninghamella echinulata* contain 92% (w/w) neutral lipids in its SCO [20]. On the other hand, another lipid producer, *Mucor circinelloides,* contained lower neutral lipid fractions (18.5%, w/w) and higher amounts of polar lipids (35%, w/w ) and free fatty acids (32% ,w/w) in its SCO [21]. In this study, the cell mass of all 5 isolates contained a high content of SCO with neutral lipid as the major fraction which is one of the desirable lipid type for biodiesel.

### Fatty acid profiles of fungal SCOs

The fatty acid composition of SCOs was analyzed for all five oleaginous isolates of mangrove fungi in order to ascertain and compare their potential as biodiesel feedstock. Alkali catalysed transesterification of these fungal SCOs resulted in fatty acid methyl esters with the profiles which are qualitatively similar but differ quantitatively among all five mangrove fungal isolates (Table 2). All the fungal SCOs in the present study were found to contain a high fraction of saturated and monounsaturated fatty acids, mainly of C_16_ and C_18_ series, which is considered a potential feature to indicate the fuel quality of fungal based diesel. This composition is quite similar to the commonly used vegetable oil feedstock for biodiesel such as rapeseed, soybean, sunflower and palm [22]. In contrast, the fatty acid profiles of oleaginous algae and cyanobacteria show a dominance of C_14_ and C_16_ fatty acids with *Chlorella* sp. being rich in C_18_[23]. The fatty acid profile of SCOs of these five mangrove fungal isolates was also found to differ from that of other oleaginous filamentous fungi. Among saturated fatty acids, palmitic acid (C16:0) was found to be present in the highest quantity with values varying between 20% (IBB M1) and 36% (IBB G4) for all the fungi. Higher amounts of stearic acid (C18:0) were also observed in the transesterified oils of all mangrove fungi with the highest content of 23.6% for isolate IBB M1 followed by 20.6% for IBB F14. High contents of stearic acid have been reported recently for *Aspergillus sp*. *viz.*, 48-57% [7] while very low contents were found in *M. circinelloides* (7%) [18] and *M. isabellina* (1%) [19]. In contrast, low quantities of palmitic acids were detected for *Aspergillus* sp [7], while it was dominant in *M. circinelloides* (20.7%) [18] and *M. isabellina* (28%) [19]. Among monounsaturated fatty acids, oleic acid (C18:1) was shown to be dominant in SCOs of all the five mangrove fungal isolates with highest content for isolate IBB G4 (41.3%) followed by the other four isolates with a content of 30 - 35%. Oleic acid contents have been found to be 0.1-1.6, 28 and 55.5% in lipids of *Aspergillus spM. circinelloides* and *M. isabellina*, respectively [7,18,19]. Fungal SCOs usually differ from most vegetable oils in being rich in PUFAs and hence are mainly exploited for PUFA production. However, PUFAs with more than 4 double bonds are not desirable for good quality biodiesel. In the present study, the major PUFA member was determined to be linoleic acid (C18:2) with contents ranging from 8.7 to 23.3% in the SCOs of all mangrove fungal isolates. The content of linolenic acid (C18:3) was found to be negligible and PUFAs with four or more double bonds, were not detected (Table 2), similar to the SCOs of *Aspergillus* sp. (0.09%) and *M. isabellina* (2.4%) [7,19]. This is in contrast with the biodiesel obtained from *M. circinelloides* and *A .terreus* MTCC 6324 wherein higher PUFA contents of C18:3 (22.5%) and C15:4, C17:4, C19:4, C32:3, C33:4 (9%), respectively were obtained [11,18].

**Table 2:** Fatty acid methyl ester composition of the SCOs of oleaginous mangrove fungal isolates

**Isolate**	FAME composition (% of total fatty acid)^*^																			
	**C8:0**	**C10:0**	**C12:0**	**C14:0**	**C15:0**	**C16:0**	**C16:1**	**C17:0**	**C17:1**	**C18:0**	**C18:1**	**C18:2**	**C18:3**	**C20:0**	**C20:1**	**C20:3**	**C20:5**	**C22:0**	**C24:0**	**T**_**FA**_
IBB B2	0.2	ND	0.2 ^a^	0.5 ^ac^	0.2	21^ab^	2.8 ^a^	0.3 ^a^	ND	13.6 ^ab^	34.5 ^a^	23.3 ^a^	0.5 ^a^	0.7 ^ab^	0.1 ^a^	0.3 ^a^	0.1 ^a^	0.8 ^a^	0.9 ^a^	100
IBB F14	ND	ND	0.4 ^b^	0.4 ^ab^	ND	29.2 ^bc^	0.2 ^b^	ND	ND	20.6 ^bc^	32.5 ^a^	14.7 ^b^	ND	1.1 ^bc^	ND	ND	ND	0.5 ^a^	0.4 ^a^	100
IBB G4	ND	ND	ND	0.6 ^c^	ND	36 ^c^	1 ^b^	ND	ND	10.7 ^a^	41.3 ^a^	8.7 ^b^	0.1 ^b^	0.5 ^a^	0.1 ^a^	ND	0.2 ^a^	0.5 ^a^	0.2 ^a^	99.9
IBB G5	ND	ND	ND	0.6 ^c^	ND	24.3 ^ab^	1.1 ^ab^	0.5 ^a^	ND	15.7 ^ab^	31.8 ^a^	21.70 ^a^	0.6 ^a^	1.4 ^c^	ND	1 ^a^	ND	0.6 ^a^	0.7 ^a^	100
IBB M1	ND	ND	ND	0.3 ^b^	ND	20.1 ^a^	0.4 ^b^	0.5 ^a^	ND	23.6 ^c^	30.1 ^a^	22.3 ^a^	0.4 ^ab^	0.8 ^ab^	0.1 ^a^	ND	0.3 ^a^	0.4 ^a^	0.7 ^a^	100

The values are means of three independent determinations. ^a,b,c^ Mean values within a column with different superscript letters differ significantly and were determined using one way ANOVA followed by Tukey and/or t-test as mentioned in Methods. Differences were considered statistically significant for p < 0.05, n = 3. Separate analysis was done for each column.

^*^ C14:0 -Myristate C16:1-Palmitoleate C18:1- Oleate C18:3-Linolenate C22:0-Behenate.

C16:0 -Palmitate C18:0-Stearate C18:2-Linoleate C20:0-Arachidate C24:0-Lignocerate.

ND not detected.

T_FA_ Total weight % of FAMEs detected and quantified by GC-FID.

### Biodiesel properties of transesterified fungal SCOs (FAMEs)

Direct measurement of fuel properties of biodiesel is quite complex with high cost, high error in reproducibility and requiring a considerable amount of fuel sample [24]. Therefore, prediction models and mathematical equations have been developed to predict biodiesel properties from FAME composition [24-30]. In the present study, the different physicochemical biodiesel properties were determined for all five SCOs using these models and/or equations based on FAME profiles as well as carried out experimentally and the results are summarized in Table 3. Among physical properties of biodiesel, the density values as determined both experimentally and from prediction models were found to be in the range for IBB M1, G4 and F14 as per the standard norms. Values for IBB B2 and G5 were below the lower limit and therefore, these isolates cannot be considered suitable for biodiesel production based on the fuel density values. The calculated kinematic viscosity values (4.52 - 4.69) at 40°C for all fungal samples were in the range of the biodiesel standard specifications.

**Table 3:** Biodiesel properties of the transesterified SCOs of oleaginous fungi from mangrove wetlands

**Property**	**Isolates**					**US biodiesel standard ASTM D6751**	**EU biodiesel standard EN 14214**	**Indian biodiesel standard IS 15607**
	**IBB B2**	**IBB F14**	**IBB G4**	**IBB G5**	**IBB M1**			
Density ^*^ (g.cm^-3^)	0.825 ^a^	0.870 ^b^	0.860 ^b^	0.810 ^a^	0.875 ^b^	NS	0.86 – 0.90	0.86 – 0.90
	(0.857)	(0.865)	(0.865)	(0.855)	(0.865)			
Kinematic viscosity^#^ (40 °C; mm^2^s^-1^)	4.52	4.69	4.55	4.55	4.68	1.9 – 6.0	3.5 – 5.0	3.5 – 5.0
SN ^*^	195 ^a^	217 ^b^	201 ^a^	190 ^a^	200 ^a^	NS	NS	NS
	(193.2)	(193.2)	(195.3)	(193.0)	(192.1)			
IV ^*^	91.5 ^a^	60.28 ^b^	73.0 ^c^	76.30 ^c^	54.81 ^b^	NS	120max	NS
	(74.9)	(53.4)	(52.5)	(69.7)	(67)			
HHV (MJ kg ^-1^)^**^	40.06	39.63	40.09	40.49	40.40	NS	NS	NS
	(40.38)	(40.7)	(40.6)	(40.47)	(40.55)			
CN ^#^	56.22	61.24	61.2	57.815	58.42	47-65	51 min	51 min
TAN (mg KOH/g) ^*^	ND	ND	0.2 ^a^	0.1 ^b^	0.1 ^b^	0.8 max	0.5 max	0.5 max
FFA (%)^*^	ND	ND	0.1 ^a^	0.05 ^a^	0.05 ^a^	NS	NS	NS
Concentration of linolenic acid (C18:3) (%) ^*^	0.5 ^a^	ND	0.1 ^b^	0.6 ^a^	0.4 ^ab^	NS	12 max	NS
FAME having ≥4 double bonds (%)^*^	ND	ND	ND	ND	ND	NS	1 max	NS

The experimental values are means of three independent determinations. ^a,b,c^ Mean values within a row with different superscript letters differ significantly and were determined as mentioned in Methods (ANOVA, Tukey, p < 0.05). Separate analysis was done for each row.

ND not detected; NS not specified.

^*^ Experimentally determined values followed by predicted ones, if any, in brackets.

^#^ Predicted values as mentioned in Methods.

^**^ Calculated using experimental SN and IV while the values in brackets determined from predicted ones.

Iodine value (IV), saponification number (SN) and higher heating value (HHV) are three important chemical properties of biodiesel attributed to the fatty acid profile. The IV is a crude measure of degree of unsaturation of the biodiesel and is often used in connection with its oxidative stability. The SN indicates the amount of TAG present in total lipid and HHV depends upon both IV and SN. Therefore, in the present study the SN and IV were experimentally determined as well as calculated empirically from fatty ester composition of transesterified total lipids. The experimentally determined and the predicted IVs were below the EN14214 specification (120 max) and suggest good oxidative stability of the transesterified oils from mangrove fungi. The calculated and experimentally determined SNs were found to be comparable for all the isolates while HHVs of ~ 40 MJ kg^-1^ were similar to methyl esters of vegetable oils [22].

For biodiesel, CN has been found to increase with an increasing weight percentage of saturated and long chain fatty ester. In fact, methyl esters of stearic acid (C18:0), which is of relevance to biodiesel, have been found to possess the highest CN (> 80) [31,32]. In the present study, methyl esters of long chain saturated fatty acids namely stearic acid (C18:0) and palmitic acid (C16:0) were found to be the major components in all transesterified fungal oils. The calculated CNs for all the mangrove isolates were found to be between 56-61(Table 3), and comparable with the predicted values reported for biodiesel obtained from other oleaginous fungi [19]. These values are in the acceptable range of international biodiesel standard norms suggesting their possible suitability as a fuel.

The total acid number (TAN) as determined according to EN14214 was estimated to be 0.1-0.2 mg KOH g^-1^ for isolates IBB G4, G5 and M1 while it was negligible in IBB B2 and F14 and were in accordance with the biodiesel standards. These TAN values were much lower as compared to biodiesel from vegetable oils which lie between 0.08-0.62 mg KOH g^-1^[22] and algal oils, (0.37-0.71 mg KOH g^-1^) [33]. Free fatty acid (FFA) content determined as per EN 14214 was found to be < 0.1% for all 5 isolates. Other chemical properties of biodiesel evaluated were the concentration of linolenic acid (C18:3) and wt % of FAMEs having ≥ 4 double bonds. From the fatty acid profiles of fungal SCOs, it can be seen that the concentration of C18:3 were well below the specified limit of 12 max and fatty esters with ≥ 4 double bonds were not detected in transesterified oils of mangrove fungi (Tables 2 and 3).

### Effect of different glucose concentrations and cheap renewable substrates on SCO yields of IBB M1

IBB M1 was selected for further studies being the highest lipid yielding strain with higher level of long chain saturated and monounsaturated FAMEs. Glucose concentration has a significant effect on cell growth and lipid accumulation in batch cultures. It is also known that lipid content is not constant during the cultivation and tends to accumulate only till the carbon source is available [8]. Hence, in order to study the effect of carbon substrate concentration on the biomass and SCO yields of strain IBB M1, batch experiments were performed in shake flasks with initial glucose concentrations ranging from 10 to 100 g L^-1^. Residual glucose concentrations were also determined to check if the culture exhausted the carbon source during the incubation period. The cellular biomass (CDW) and lipid yield increased upto 5.5 and 3.0 g L^-1^ at 30 g L^-1^ glucose, with both biomass and lipid yields decreasing beyond 50 g L^-1^ (Table 4), indicating an inhibitory effect of glucose. The lipid content was maximal at 30 g L^-1^ (0.54 g g^-1^) which decreased to 0.48 g g^-1^ CDW at 100 g L^-1^ glucose. The glucose source was almost consumed by 72 h for all the initial glucose concentrations studied up to 100 g L^-1^ as evident by the residual glucose levels shown in Table 4. Thus, this data suggests that for IBB M1, maximal biomass and lipid yield were obtained at 30 g L^-1^ of initial glucose concentration. Similar results have been observed for the oleaginous yeast *Rhodosporidium toruloides* Y4 and *Cryptococcus curvatus* O3, wherein biomass and lipid production were found to be maximal at glucose concentrations up to 90 g L^-1^ and 120 g L^-1^, respectively. For both these yeasts, biomass and lipid yields were found to decrease beyond these substrate concentrations, suggesting an inhibitory effect of glucose [34,35].

**Table 4:** Growth and lipid (SCO) yields by strain IBB M1 at differing initial glucose concentrations

**Glucose (g L**^**-1**^**)**	**Biomass (CDW in g L**^**-1**^)*	**Lipid yield (g L**^**-1**^**)**	**Lipid content (g g**^**-1**^of CDW )^*^	**Residual glucose (g L**^**-1**^**)**	**Lipid/glucose yield coefficient (g g**^**-1**^**)**
10	4.6 ± 0.3 ^a^	2.28 ± 0.09 ^a^	0.49 ± 0.03 ^a^	0.16 ± 0.02^a^	0.23 ± 0.01 ^a^
30	5.5 ± 0.1 ^b^	3.00 ± 0.13 ^b^	0.54 ± 0.02 ^a^	0.18 ±0.01 ^ab^	0.10 ± 0.03 ^b^
50	5.3 ± 0.2 ^bc^	2.60 ± 0.1 ^a^	0.49 ± 0.02 ^a^	0.18 ±0.01 ^ab^	0.05 ± 0.01^c^
70	4.9 ± 0.1 ^ac^	2.35 ± 0.08 ^a^	0.48 ± 0.05 ^a^	0.19 ± 0.009 ^b^	0.03 ± 0.009 ^c^
100	4.0 ± 0.3 ^d^	1.93 ± 0.2 ^c^	0.48 ± 0.04 ^a^	0.25 ±0.014 ^c^	0.02 ± 0.01 ^c^

The data are shown as the mean ± standard deviation (n = 3). ^a,b,c^ Mean values within a column with different superscript letters differ significantly were determined as mentioned in Methods (ANOVA, Tukey test). Differences were considered statistically significant for p < 0.05. Separate analysis was done for each column.

^*^ CDW- Cell Dry Weight.

The ability of strain IBB M1 to accumulate lipids on locally available cheap carbon sources was also evaluated. The agro-residues, sugarcane bagasse, grape stalks and groundnut shell are the major lignocellulosic materials in tropical countries such as India and are readily available. Cheese whey as another commonly available cheap renewable substrate was also tested. Preliminary studies showed that the lipid yields of IBB M1 when cultivated using milled grape stalk, groundnut shell, sugarcane bagasse (1%, w/v) and cheese whey were 3.62, 10.56, 16.63 g 100 g^-1^ DW of substrate and 13.1 g 100 g^-1^ CDW respectively after 72 h. Thus IBB M1 was able to accumulate SCO to varying degrees on these cheap renewable substrates. To date, to the best of our knowledge, no known reports on milled sugarcane bagasse, grape stalk and groundnut shell for SCO production by filamentous fungi exist. Subramaniam *et al* (2010) have reviewed other low cost feedstocks for microbial lipid production which include industrial glycerol, corn steep liquor, molasses, sweet sorghum, municipal wastewater and effluents [4]. Recently an oleaginous yeast *Yarrowia lipolytica* has been shown to produce SCO (58.5%) on sugarcane bagasse hydrolysate medium [36] while Zygomycetous fungi have been shown to produce significant quantities of biomass and SCO when grown on cheese whey [37]. Thus, the lipid yields obtained on the cheap substrates evaluated in the present study are of importance as preliminary data indicates that IBB M1 was able to utilize these lignocellulosic wastes for SCO production.

### Identification of fungal isolate IBB M1

**Figure 3:**
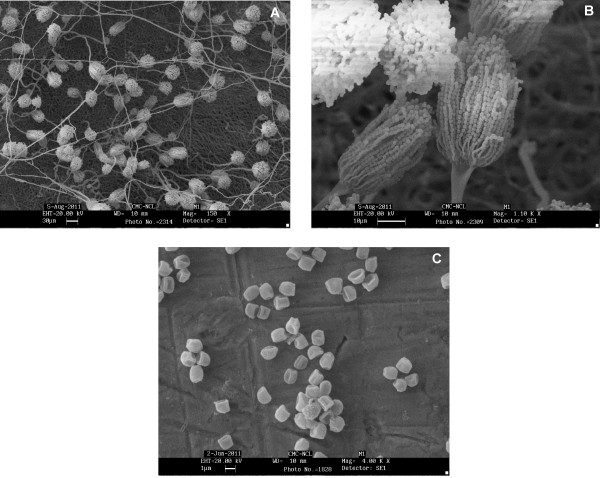
**Scanning electron microscopy of fungal isolate*Aspergillus terreus*IBB M1 showing morphological features.A**) Surface of the fungal colony showing conidiophores arising from the basal network of vegetative hyphae at 150X (Bar indicates 30 μm); **B**) Conidial heads bearing conidia in compact columnar arrangement at 1.10 K X (Bar indicates 10 μm); **C**) Conidia at 4.0 K X (Bar indicates 1 μm).

Based on morphological characteristics and with the help of an identification key [38], the isolate IBB M1 was classified as species of *Aspergillus terreus* gr. Cinnamon coloured colonies on standard culture media, compactly columnar conidial heads as revealed in SEM analysis (Figure 3) and the presence of accessory conidia on hyphae were among the key features to identify the isolate IBB M1 as a strain of *Aspergillus terreus*. Further, 18 S rRNA gene sequencing was used to confirm the identity. The PCR amplification of the genomic DNA using universal fungal primer set NS1/NS4 yielded a 1.1 KB DNA fragment. Sequence data of 1038 nucleotides was obtained from DNA sequencing and submitted to GenBank [GenBank: JN639854]. After BLAST analysis, IBB M1- specific amplicons showed 100% identity with *Aspergillus terreus* [GenBank:AF516138.1; 18 S rRNA gene partial sequence] and *Aspergillus terreus* [GenBank:AB008409.1; gene for 18 S rRNA partial sequence]. Thus, the isolate IBB M1 was identified as a strain of *Aspergillus terreus* on the basis of both morphological and molecular studies.

An earlier report by Singh and Sood [39] suggested that *Aspergillus terreus* Thom 309 had high SCO producing ability, but to date has been not explored for its biodiesel potential. A total lipid content of 51% (w/w) has been reported for *A. terreus* Thom 309 similar to that of IBB M1 but with a different FAME profile. *A. terreus* IBB M1 exhibited suitable fatty acid profiles for biodiesel with high stearic acid content (23.6%) and low linolenic acid content (0.4%) while the earlier report (*A. terreus* Thom 309) showed 0.3% and 20.7%, respectively. Higher contents of long chain monounsaturated fatty acid esters and negligible amounts of PUFAs are desirable for fuel properties such as CN and IV specified in biodiesel standards and these criteria seem to be fulfilled by IBB M1.

## Conclusion

In the present study, the SCOs of fungal cell mass from the mangrove wetlands of Indian west coast were explored as biodiesel feedstock. Five isolates of oleaginous filamentous fungi were obtained with the neutral lipid as the major component of their total lipids. The presence of higher quantities of saturated and monounsaturated C_16_ and C_18_ fatty acids and the absence of long chain PUFAs were the major features of the SCOs of these tropical mangrove fungi. The experimentally determined and predicted biodiesel properties based on FAME composition of the fungal SCOs of 3 isolates are found to lie within the range specified by international biodiesel standard specifications. *Aspergillus terreus* IBB M1 with the highest yield of SCO was identified as a promising strain for further studies. Preliminary studies suggest that *Aspergillus terreus* IBB M1 from the mangrove wetlands is able to utilize cheap renewable substrates such as cheese whey and agro-residues *viz*., sugarcane bagasse, grape stalk and groundnut shell for SCO production. It is currently being studied further with respect to utilization of agricultural residues in order to optimize the productivity of cell mass rich in SCO. Thus, oleaginous fungi from the mangrove ecosystem with their varied lignocellulolytic enzymes could play a key role in the overall economics of biodiesel production.

## Methods

### Materials

Analytical grade solvents and chemicals *viz*., chloroform, methanol, acetone, formaldehyde, KH_2_PO_4_, Na_2_HPO_4_.12H_2_O, anhydrous sodium sulphate, NaCl, KCl, and MgCl_2_ were purchased from Merck Ltd., Mumbai, India. Czapek-Dox medium was obtained from HiMedia laboratories, Mumbai, India while 1, 1, 1-trichloroethane, from National Chemicals, Vadodara, India. Silicic acid, chromatography grade and Nile red were purchased from Sigma-Aldrich, Inc., USA. ‘Bead beater’ was procured from Biospec Products, Inc., USA. TAN and FFA content were determined using Kittiwake DIGI Biodiesel test kit, purchased from Kittiwake Developments Ltd., UK. Iodine solution (Wijs) was purchased from Acros Organics, Belgium while alcoholic KOH solution was procured from Merck, Germany. Potassium iodide and phenolphthalein were obtained from Fisher Scientific, Mumbai, India. All other chemicals obtained locally were of AR grade and at least 98% pure according to the manufacturer.

### Sampling sites and isolation of mangrove fungi

The present study was carried out on 14 fungal strains which were isolated from mangrove wetlands of the Indian west coast located at Bandra in Mumbai (19°3′N latitude and 72°49′E longitude), Murud (18°18′N latitude and 72°58′E longitude) and Mandovi estuary in Goa (15°29′N latitude and 73°50′E longitude). The sampling was done in December 2007 and May 2008. The soil samples rich in detritus material were collected from the base of the mangrove trees in sterile 50 mL screw-cap tubes. The soil samples (1 g) were weighed and inoculated into 50 mL of Czapek Dox broth and incubated with shaking (180 rpm) for 72 h at 28°C. After incubation, this broth was used to streak Czapek Dox agar plates to obtain the isolates. Penicillin and streptomycin were added to the media at concentrations of 400 U mL^-1^ and 1mg L^-1^, respectively, to prevent bacterial growth.

### Media and incubation conditions

Czapek Dox basal medium used for isolation contained (gL^-1^ ), 30.0 sucrose, 3.0 NaNO_3_, 1.0 K_2_HPO_4_, 0.5 MgSO_4_.7H_2_O, 0.5 KCl and 0.01 of ferrous sulphate supplemented with 15 g L^-1^ of NaCl, 6.7 mg L^-1^ of ZnSO_4_.7H_2_O and 1 mg L^-1^ Co[NO_3__2_.6H_2_O. Isolated fungal strains were maintained on the above Czapek Dox agar slants at 4°C. The lipid fermentation medium used during all screening stages of the mangrove fungal isolates was carbon rich and nitrogen limited to induce lipid accumulation and contained (g L^-1^), 30.0 glucose, 1.5 yeast extract, 15.0 NaCl, 0.5 NH_4_Cl, 5.0 Na_2_HPO_4_.12H_2_O, 7.0 KH_2_PO_4_, 1.5 MgSO_4_.7H_2_O, 0.1 CaCl_2_.2H_2_O, 0.01 ZnSO_4_.7H_2_O, 0.08 FeCl_3_.6H_2_O, 0.1 CuSO_4_.5H_2_O, and in mg L^-1^, 0.1 Co[NO_3__2_.6H_2_O, 0.1 MnSO_4_.5H_2_O and pH adjusted to 5.5 [15]. The abovementioned media was inoculated with 1 x10^6^-10^8^spores mL ^-1^ and incubated on a rotary shaker (120 rpm) at 30°C for 72 h. The fungal cell mass was harvested by filtration and washed thrice with distilled water.

Batch experiments were performed in conical flasks in the abovementioned medium containing initial glucose concentrations ranging from 10–100 g L^-1^ as well as on locally available cheap waste substrates *viz.*, cheese whey and agro-residues including sugarcane bagasse, ground nut shell and grape stalks. The agro-residues were washed, dried, milled to pass through a 1 mm sieve and added to the medium as a sole carbon and energy source (1% w/v). All other cultivation and incubation conditions were the same as mentioned above.

### Nile red staining

All the fungal strains were checked for intracellular LBs indicative of lipid accumulation by Nile red fluorescence staining[15,40]. Microscopy was performed with a Nikon Eclipse 80i light microscope, equipped with a digital camera using a 465–495 nm excitation filter, a 505 nm diachronic mirror and a 515–535 nm barrier filter.

### Extraction and gravimetric determination of total lipids as SCO

The harvested and washed cell mass was freeze dried. The dried biomass was crushed to powder, the dry weight (g) measured gravimetrically and used for lipid extraction which was performed according to Schneiter and Daum (2006) after cell disruption [41]. Briefly, two techniques were used for disruption of fungal cells in order to determine optimal cell lysis for extraction of intracellular lipids. The first technique employed a ‘bead beater’ and the second one used liquid nitrogen. For bead beater extraction, a known dry weight of fungal biomass was transferred to a 20 mL chamber of the bead-beater filled up to its 3/4^th^ volume with 0.5 mm acid washed glass beads. Methanol (10 mL) was added to fill the rest of the chamber and 9–20 bead beating cycles (each of 1 min) were carried out in cold conditions till cell breakage was confirmed by microscopy. For disruption by liquid nitrogen, dry biomass sample was transferred to a chilled mortar pestle containing glass beads to which 10 mL methanol was added and the sample finely ground. In both the techniques, the homogenate obtained after cell disruption was collected, transferred to glass stoppered flask and processed [41]. The final extracted organic phase obtained was transferred to a pre-weighed round bottom flask and evaporated in a rotary evaporator at 55°C under vacuum. The flask containing total lipid extract was weighed and the total lipid extract as SCO was gravimetrically determined. The total lipid extract was reconstituted in 5–6 mL chloroform / methanol (2:1, v/v) and stored in clear screw top glass vials at −20°C till further use.

### Fractionation of SCOs

The total neutral lipid content in the extracted SCOs of five oleaginous fungal isolates was determined by lipid fractionation [42]. Briefly, a known weight of total lipid extract was dissolved in chloroform (1 mL) and fractionated using a column (25 mm x 100 mm) of silicic acid (1 g), activated by heating overnight at 110°C. Successive applications of 1,1,1- trichloroethane (100 mL) , acetone (100 mL) and methanol (50 mL) were carried out for elution of neutral lipids, glycolipids plus sphingolipids and phospholipids, respectively. The weight of each fraction was determined after evaporation of the respective solvent.

### Preparation and analysis of FAMEs from fungal SCOs

To analyze the fatty acid profile of the SCOs of oleaginous tropical mangrove fungi, the transesterification was carried out according to Leung *et al.* (2010) [22]. The reaction was carried out in a 50 mL round bottom flask kept in a thermostatic bath with a reflux condenser and a magnetic stirrer using a methanol to oil molar ratio of 60:1 and a catalyst (NaOH) concentration of 1.5-3 wt % relative to SCO. The reaction was carried out under ambient pressure for 90 minutes at 60°C. The mixture was allowed to stand for 1 h to collect the upper organic layer (FAMEs) and the solvent was removed by rotary evaporator (Büchi, Rotavapor RE120, 70°C). The FAME samples were reconstituted in chloroform/ methanol (2:1, v/v) and stored in clear screw top glass vials at −20°C till further use.

To obtain the fatty acid profile of the transesterified fungal SCOs, all the samples were appropriately diluted with chloroform/ methanol (2:1, v/v) mixture and the sample analyzed using gas chromatograph as per the AOAC standard method [43]. A capillary column CP-Sil88 of 0.25 mm ID and 50 m length with flame ionization detector (GC1000, Chemito) was used. The column temperature was programmed as being upgraded from 100°C to 198°C at a rate of 1.5°C /min and held for 8 min. Nitrogen was used as the carrier gas. The injector and detector temperatures were set at 225°C and 250°C, respectively. Identification of peaks was performed by comparison with authentic FAME standard mixture and quantifications done on the basis of their specific peak areas.

### Determination of biodiesel properties of SCOs

Different physicochemical fuel properties namely density, kinematic viscosity, SN, IV, HHV, CN, TAN and FFA were determined experimentally as well as by using predictive models and mathematical equations for the transesterified SCOs of the five oleaginous fungal isolates.

Density values were experimentally determined using a pycnometer (10 ml ) and also estimated by predictive model using equation (1) based on Kay’s mixing rule [25]. The density database of individual pure fatty acid methyl ester compounds [26] was used for calculations.

(1) $$\rho =\sum ({\text{c}}_{i}\rho_{i})$$

where c_i_ is the concentration (mass fraction) and ρ_i_ is the density of component (individual fatty acid methyl ester) present in biodiesel.

Kinematic viscosity values (40°C; mm^2^ s^-1^) were calculated using equation (2), a modified Grunberg-Nissan equation for biodiesel [27]

(2) $$\nu_{\text{mix}}=\sum {\text{A}}_{\text{c}}\times \nu_{\text{c}}$$

where ν _mix_ = the kinematic viscosity of the biodiesel sample (mixture of fatty acid alkyl esters), A_c_ = the relative amount (%/100) of the individual neat ester in the mixture (as determined by, GC-FID) and v_c_ = obtained from the kinematic viscosity database of individual FAME present in biodiesel [27].

SN and IV were determined experimentally [44] and calculated empirically from FAME composition, with the help of Eq. (3) and (4), respectively:

(3) $$\text{SN}=\sum (560\times {\text{A}}_{\text{i}})/{\text{MW}}_{\text{i}}$$

(4) $$\text{IV}=\sum (254\times \text{D}\times {\text{A}}_{\text{i}})/{\text{MW}}_{\text{i}}$$

where, A_i_ is the percentage, D is the number of double bonds and MW_i_ is the molecular mass of each fatty acid methyl ester [28,29].

The higher heating value (HHV) also known as gross heat of combustion or heat content of an oil or biodiesel depends on the SN and IV of that fuel and therefore, for calculation of HHVs (expressed as MJ kg ^-1^), Eq. (5) has been used [30].

(5) $$\text{HHV}=49.43-\left[0.041\left(\text{SN}\right)+0.015\left(\text{IV}\right)\right]$$

Cetane Number (CN) was calculated using the multiple linear regression equation Eq. (6) [24]:

(6) $$\text{CN}=1.068\sum \left({\text{CN}}_{\text{i}}{\text{W}}_{\text{i}}\right)-6.747$$

where CNi represent reported CN of pure fatty acid methyl ester available in database[24] and W_i_ is the mass fraction of individual fatty ester component detected and quantified by GC-FID.

### Identification of fungal isolate IBB M1

The filamentous fungal isolate IBB M1 was identified in the laboratory by both morphological and molecular approaches. Identification was based on macroscopic observation of the colonies and examination of the microstructural characteristics using universal identification keys for fungi [38]. Scanning electron microscopy analysis was carried out to study the microscopic characteristics (Stereoscan 440, LEO/Leica, Cambridge, UK).

In molecular techniques for identification, genomic DNA of the fungal strain IBB M1 was isolated by using the standard protocol [45]. Partial region of SSU rDNA was amplified by PCR using universal fungal primers, NS1 (5’-GTAGTCATATGCTTGTCTC-3’) and NS4 (5’-CTTCCGTCAATTCCTTTAAG-3’) of 1,100 bp [46]. PCR products were purified by using gel extraction kit (GeNei, Bangalore, India) and sequenced using the Big Dye Terminator cycle sequencing kit (Applied Biosystems, USA) according to the manufacturer’s protocol followed by purification using Big Dye X-Terminator Purification kit (Applied Biosystems, USA) and analyzed in a DNA Analyzer (3730 DNA Analyzer, Applied Biosystems, USA). Sequence data were analysed using Sequence analysis software. The 18 S sequence obtained was aligned using BLAST algorithm to find matches within the non redundant database at NCBI (National Centre for Biotechnology Information; http://blast.ncbi.nlm.nih.gov/Blast.cgi) [47]. Sequence data were submitted to GenBank through submission tool BankIt of NCBI.

### Statistical analysis

All values are means of three independent experiments. Statistical analyses were performed using SPSS 11.5 statistics software (SPSS Inc., Chicago, IL, USA). Means were compared and analyzed using either t-test or one-way analysis of variance (ANOVA) with Tukey HSD *post hoc* multiple comparison test. Differences were considered statistically significant for p < 0.05.

## Abbreviations

CDW, Cell Dry Weight; CN, Cetane Number; FAME, Fatty Acid Methyl Ester; FFA, Free Fatty Acid; GC-FID, Gas Chromatography Flame Ionization Detector; HHV, Higher Heating Value; IV, Iodine Value; PUFA, Polyunsaturated Fatty Acid; SCO, Single Cell Oil; SN, Saponification Number; TAG, Triacylglycerol; TAN, Total Acid Number.

## Competing interests

The authors declare that they have no competing interests.

## Authors’ contributions

MK performed the experiments as a part of his doctoral work. SK helped in the isolation, maintenance, growth of cultures and sequence analysis.SSZ participated in the statistical analysis of the data; AP participated in the design of experiments and helped to draft the manuscript.BAC helped in the sequence analysis. The work was carried out under the supervision of ARK who conceived and coordinated the study, designed the experiments and helped to draft the manuscript. All the authors have read and approved the final manuscript.
